# Effect of supervised exercise in groups on psychological well-being among pregnant women at risk of depression (the EWE Study): study protocol for a randomized controlled trial

**DOI:** 10.1186/s13063-017-1938-z

**Published:** 2017-05-05

**Authors:** Lotte Broberg, Mette Backhausen, Peter Damm, Per Bech, Ann Tabor, Hanne Kristine Hegaard

**Affiliations:** 1 Research Unit Women’s and Children’s Health, Juliane Marie Centre for Women, Children and Reproduction, Copenhagen University Hospital, Rigshospitalet, Copenhagen, Denmark; 2 Department of Obstetrics, Copenhagen University Hospital, Rigshospitalet, Blegdamsvej 9, 2100 Copenhagen, Denmark; 3 Institute of Clinical Medicine, Faculty of Health and Medical Sciences, University of Copenhagen, Copenhagen, Denmark; 4 Psychiatric Research Unit, Psychiatric Centre North Zealand, Copenhagen University Hospital, Hillerød, Denmark; 5 Center of Fetal Medicine, Department of Obstetrics, Copenhagen University Hospital, Rigshospitalet, Copenhagen, Denmark; 6 Department of Health Sciences, Faculty of Medicine, Lund University, Lund, Sweden

**Keywords:** Pregnancy, Antenatal, Supervised exercise, Depression, Anxiety, Psychological well-being, Randomized controlled trial

## Abstract

**Background:**

Pregnant women with depression and/or anxiety prior to pregnancy are at higher risk of preterm birth, breastfeeding problems, postpartum depression, and disruption of the mother-infant attachment. It is well documented that exercise improves psychological well-being in nonpregnant subjects with symptoms of depression. However, in only a few small studies have researchers examined the effect of exercise on symptoms of depression among pregnant women. We hypothesize that physiotherapist-supervised group exercise for pregnant women at risk of antenatal depression increases their psychological well-being. This paper describes the study protocol of a randomized controlled trial (RCT) on a supervised group exercise intervention for pregnant women with a current or previous history of depression and/or anxiety.

**Methods/design:**

The RCT is being carried out at the Department of Obstetrics, Rigshospitalet, Copenhagen University Hospital, in the period 2016–2019. The inclusion criteria are pregnant women ≥18 years of age with depression and/or anxiety requiring treatment by a psychiatrist or a psychologist within the last 10 years and/or intake of antidepressants in the 3 months prior to conception and/or during pregnancy. The women must have appropriate Danish language skills, be pregnant with a single fetus, give written informed consent, and be at 17–22 gestational weeks when the intervention begins. The primary outcome is psychological well-being (the five-item World Health Organization Well-being Index). Secondary outcomes are symptoms of depression (Edinburgh Postnatal Depression Scale), functional ability (General Health Questionnaire), clinical symptoms of anxiety (State-Trait Anxiety Inventory), sleep quality and sleep disturbances (Pittsburgh Sleep Quality Index), and pregnancy and delivery outcomes. The intervention is supervised group exercise twice weekly for 12 weeks. The control group will receive standard antenatal care. On the basis of sample size calculation, a total of 300 women will be randomly assigned to either the intervention or the control group in a ratio of 1:1.

**Discussion:**

The trial is expected to contribute to the body of knowledge used in planning antenatal care for pregnant women at risk of depression.

**Trial registration:**

ClinicalTrials.gov, NCT02833519. Registered on 19 May 2016.

**Electronic supplementary material:**

The online version of this article (doi:10.1186/s13063-017-1938-z) contains supplementary material, which is available to authorized users.

## Background

Mental illness affects individuals’ functioning, resulting in emotional suffering and reduced quality of life for the affected persons as well as social and economic consequences for society [1]. A reduction in mental illness is of great importance for public health [2], and the World Health Organization (WHO) has emphasized this by giving greater attention to the prevention of mental illness and the promotion of mental health in health care systems [1]. Among other initiatives, the WHO has taken an initiative to unite knowledge and forces across national borders to reach consensus regarding which activities would be appropriate to increasing the quality of mental health care, including specific steps to be taken [1].

Maternal depression and anxiety are the most common psychiatric disorders during pregnancy and in the postpartum period [3]. A systematic review based on 28 studies done in a wide range of developed countries estimated the prevalence of antenatal depression to range from 6.5% to 12.9% and the prevalence of depression during the first 3 months postpartum to be up to 19% [4]. Antenatal anxiety is reported in 4% to 39% of all pregnant women and in up to 16% of all women in the postpartum period [5].

The consequences of depression and anxiety in the antenatal and postnatal period are extensive [6–13]. Women with antenatal depression have an increased risk of preterm birth [6] and postpartum depression [7], as well as a lesser likelihood of breastfeeding initiation [6]. Antenatal and postnatal depression is associated with disruption of the mother-infant relationship [8, 9], developmental delays in childhood [10], and behavior problems such as low adaptive functioning and social competence in childhood and adolescence [11, 12]. Antenatal anxiety is associated with an increased risk of low birth weight [13] and increased risk of attention and hyperactivity problems, as well as other behavioral and emotional challenges, in childhood [14, 15].

The strongest risk factor for antenatal depression and anxiety is a history of depression and/or anxiety prior to pregnancy [16]. About 20% of women with a history of depression prior to pregnancy experience recurrent major depression during pregnancy or postpartum, and 56% of women with anxiety prior to pregnancy experience anxiety in this period. Among women with comorbid depression and anxiety prior to pregnancy, 29% report a recurrent major depressive episode, and as many as 63% experience anxiety during pregnancy and the postpartum period [16].

The major challenges outlined have led to both national and international guidelines recommending a coordinated care plan for this group of pregnant women [17, 18]. This plan is recommended to include screening and treatment for depression and anxiety as part of routine antenatal care, including interdisciplinary and intersectoral teamwork with obstetricians, psychiatrists, psychologists, social workers, nurses, and midwives [17, 18].

Randomized studies have shown that exercise among clinically depressed patients has a positive effect on signs of depression and anxiety [19]. However, until now, in very few studies have researchers examined the effects of exercise in the prevention of or treatment for symptoms of depression and anxiety among pregnant women. According to a meta-analysis by Daley et al. [20], there is some evidence that exercise may be effective in treating antenatal depression, but as the authors point out, this conclusion is based on trials with low to moderate methodological quality and on a small meta-analysis sample size. Their meta-analysis also concluded that more high-quality trials are needed to examine whether exercise is effective in treating signs of depression during pregnancy.

The Effect of Group Exercise on Mental Wellbeing among Pregnant Women at Risk of Perinatal Depression trial (EWE Study) is a large randomized controlled trial (RCT) using supervised exercise in groups twice weekly for 12 weeks as an intervention for pregnant women at risk of depression and anxiety. The intervention period is 12 weeks because a major meta-analysis based on 49 studies of exercise and depression found that a training period of 10–16 weeks had the best effect on symptoms of depression [19]. Supervised exercise in groups was chosen because pregnant women experience a sense of security when they exercise together in professionally led sessions where the professional can monitor the extent of the physical exercise and provide guidance [21].

### Objective

The aim of this RCT is to assess whether a supervised group exercise intervention increases psychological well-being in pregnant women with a current or previous history of depression and/or anxiety. This paper describes the design of the study.

## Methods/design

### Study design

The EWE Study is a parallel-group RCT designed to evaluate the effect of supervised exercise in groups on psychological well-being among pregnant women with a current or previous history of depression and/or anxiety. The EWE Study was designed in accordance with the Consolidated Standards of Reporting Trials (CONSORT) recommendation for RCTs [22] and with the Standard Protocol Items: Recommendations for Interventional Trials (SPIRIT) guidelines for reporting trial protocols (Additional file 1) [23].

### Study setting

Participants will be recruited among pregnant women attending antenatal care at the Department of Obstetrics, Rigshospitalet, Copenhagen University Hospital, Copenhagen, Denmark. This hospital serves as a primary birth facility for women from the city of Copenhagen, as well as being a tertiary referral center, with 5672 deliveries in 2015. The intervention will take place at Rigshospitalet, Copenhagen University Hospital.

### Inclusion criteria

Eligible participants will be pregnant women ≥18 years of age with depression and/or anxiety requiring treatment by a psychiatrist or a psychologist within the last 10 years and/or intake of antidepressants in the 3 months prior to conception and/or during pregnancy. The women must have appropriate Danish-language skills, be pregnant with a single fetus, give written informed consent, and be at 17–22 gestational weeks when the intervention begins. Pregnant women who fulfill the inclusion criteria but have a chronic disease are included in the study only after prior agreement with an obstetrician. Chronic disease refers to a diagnosis where signs, symptoms, and treatment imply an expected long duration and lack of cure, such as certain types of heart disease and diabetes [24].

Depression in this study covers the *Diagnostic and Statistical Manual of Mental Disorders, Fifth Edition* (DSM-5), criteria of disruptive mood dysregulation disorder, major depressive disorder, persistent depressive disorder (dysthymia), bipolar disorder, and unspecified depressive disorder [25]. Anxiety in this study covers the DSM-5 criteria of separation anxiety disorder, specific phobia, social anxiety disorder, panic disorder, agoraphobia, generalized anxiety disorder, and obsessive compulsive and related disorders [25].

### Exclusion criteria

Potential participants will be excluded from participation for the following reasons: age <18 years, multiple pregnancies, substance abuse problems, eating disorders, pelvic girdle syndrome (diagnosed by a physiotherapist or medical doctor) in the current or a previous pregnancy, fetal malformations (diagnosed by ultrasound), fetal chromosomal disorder, or severe obstetric or medical complications.

### Withdrawal

Participants will be withdrawn from the study after randomization if they meet one or more of the following criteria: an occurrence of pelvic instability, preeclampsia, vaginal bleeding, or symptoms of preterm labor contraindicating physical activity.

### Recruitment procedure

**Fig. 1:**
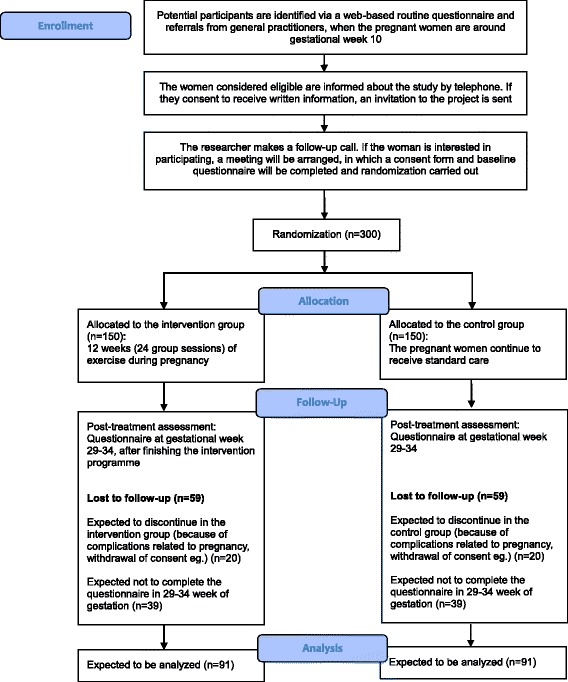
The expected flow diagram of progress through the study

As part of standard antenatal care, two midwives responsible for coordinating antenatal mental health care at the Department of Obstetrics, Rigshospitalet, Copenhagen University Hospital, will contact all pregnant women with current or previous mental health problems by telephone. Women considered eligible for the EWE Study will be made aware of the study and their opportunity to participate. Women showing interest in participating in the study and who give verbal consent to receive written information will be sent an invitation with a detailed description of the study. Approximately 2 weeks after the information has been sent, a research midwife will contact the women by telephone to ensure the invitation has been received and to answer preliminary questions. Women who are interested in further information about the study will be invited to a face-to-face meeting with a research midwife at the Department of Obstetrics, Rigshospitalet, Copenhagen University Hospital. At this meeting, the women will receive more information about the study and will have the opportunity to ask questions. If the women decide to participate, the baseline questionnaire will be completed, hand grip strength test will be measured by a North Coast Hydraulic Hand Dynamometer (North Coast Medical, Gilroy, CA, USA) [26], and randomization will be carried out (*see* Fig. 1). The participants will be randomly assigned to either the intervention or the control group in a ratio of 1:1.

### Randomization

Simple randomization will be performed on the Internet by a project midwife. The randomization code will be generated by a specially designed computer program (Lucidity Software, Melbourne, Australia) in cooperation with the Department of Clinical Medicine, The University of Aarhus, Aarhus C, Denmark.

### Blinding

Because of the nature of the intervention in this RCT, double-blinding is not possible. The statistician performing the statistical analyses will be blinded to intervention or the control group allocation.

### Sample size estimation

The sample size estimation is based on the primary outcome—psychological well-being—measured by the WHO (Five) Well-being Index (WHO-5) [27]. A sample size calculation was carried out to detect a 10-point higher score on the WHO-5 at 29–34 weeks of gestation in the intervention group compared with the control group. A 10-point difference in the WHO-5 score between the intervention and control groups is considered clinically significant [28, 29], and an SD of 16 was used. This SD is based on a previous assessment of the WHO-5 score in a population of 3970 pregnant women with and without mental disorders measured at 10–12 weeks of gestation in the total population of pregnant women at Rigshospitalet, Copenhagen University Hospital. This SD value (16 points) was used in the sample size calculation because we did not have an SD in the WHO-5 score measured at 29–34 weeks at our disposal. We expect 50% of the participants to follow the program for at least 75% of the sessions (high participation, 19–24 sessions), whereas 35% will participate in 50% to 75% of the sessions (moderate participation, 12–18 sessions) and 15% will engage in less than half of the sessions (low participation). We expect high participation in the exercise intervention to lead to an increase of 10 points in the WHO-5 score, moderate participation to lead to an increase of 7 points, and low participation to lead to an increase of 2 points compared with the control group. This will lead to the intervention group’s having an average value 7.75 points (0.50 × 10 + 0.35 × 7 + 0.15 × 2) higher than that of the control group at 29–34 weeks of gestation. With a power of 90% and a two-sided significance level of 5%, a difference in well-being level of 7.75 points (SD 16) in the two groups at 29–34 weeks of gestation can be detected by a two-sample *t* test with 91 patients in each group.

We expect that it will be necessary to include a total of 300 pregnant women because we anticipate that 13% (20 women) in each group will drop out as a result of discomfort or complications related to pregnancy and that 39 women (30%) of the remaining 131 participants in each group will not answer the questionnaire at 29–34 weeks of gestation, leaving 91 participants in each group.

### Intervention group

The intervention group will be offered an exercise program delivered to groups of 10–12 pregnant women at the hospital twice weekly for 12 weeks from baseline (17–22 weeks of gestation) in addition to standard antenatal care as described for the control group. The exercise program (*see* Table 1) will be carried out in accordance with the Danish national recommendations for exercise during pregnancy [30] and developed by physiotherapists from Rigshospitalet, Copenhagen University Hospital, who will also supervise the exercise sessions. The duration of a session will be 70 minutes, and one session will consist of a 10-minute warm-up (Borg scale 7–10); 20 minutes of endurance training on exercise bikes, treadmills, or cross-trainers (Borg scale 11–15); 25 minutes of strength training (back, abdomen, thighs, arms, and pelvic floor); and 15 minutes of stretching and relaxation (Borg scale 6) [31, 32].

**Table 1:** Intervention group exercise program

Duration	Intervention	Description of exercise	Load
10 minutes	Warm-up Up to each teacher Has to include: • Light cardiovascular exercise • Mobilization of shoulder/back/pelvis	Examples: Neck stretching: standing in a circle, perform shoulder rotations and elevations Exercise of low intensity Walking in a circle: forward, backward, and sideways Shoulder/spine/pelvic mobility 1. Arm swings (one arm + both arms) 2. Arm swings in a kayak-paddling motion 3. Lateral flexions 4. Pelvic tilts (anterior, posterior, bilateral, figure eights) 5. On all fours, flexion/extension of the spine to find the neutral position	Borg Scale 7–10
20 minutes	Endurance training	Exercise bikes, treadmills, or cross-trainers	Borg Scale 11–15
25 minutes	Strength training Pelvic floor	1. On all fours: elevation of the extremities to engage the back and hamstrings; diagonal lift, 3 × 10 repetitions followed by a 10-second isometric hold; there is a short break after each isometric hold 2. Supine position: upper extremity flexion/extension to strengthen the upper arms and pectoral muscles; 3 × 10 repetitions; 60-second break after each of the three sets 3. Squats: strengthening of the thighs and gluteal muscles; 3 × 12 repetitions; 60-second break after each of the three sets Supine position (or side-lying position if preferred) 1. Short muscle squeeze × 10 2. Squeeze held for 6–8 seconds × 20 with a 6- to 8-second break between each squeeze 3. Squeeze held for 30 seconds × 2 with a 15-second break between each squeeze	60–70% of one-repetition maximum endurance
15 minutes	Stretching and relaxation Up to each teacher Has to include: • Breathing exercises • Relaxation • Stretching	Example: Relaxation compact disc or verbal guiding with emphasis on breathing; approximately 5 minutes Stretching of the big muscle groups: • Pectoralis major • Gluteus maximus • Quadriceps • Triceps surae	Borg Scale 6

The participating women’s general practitioners will be informed about the intervention and will be encouraged to support them to continue participating in the exercise program. Furthermore, a weekly supportive email will be sent to the participants to increase compliance, and the participants in both groups will be informed that they can contact the research group if they have questions related to the trial.

### Control group

Women in the intervention group as well as those in the control group will be offered the standard antenatal care for pregnant women with a current or previous history of depression and/or anxiety. Standard care for this group covers a coordinated program including more frequent and lengthier prenatal consultations with specialized midwives and obstetricians who have particular experience with pregnancy and mental disorders. If the pregnant women are treated with antidepressants, additional ultrasound scans to assess fetal growth will be recommended. The specialized midwife coordinates the teamwork with obstetricians, psychiatrists, psychologists, social workers, and nurses.

### Outcome measures

**Fig. 2:**
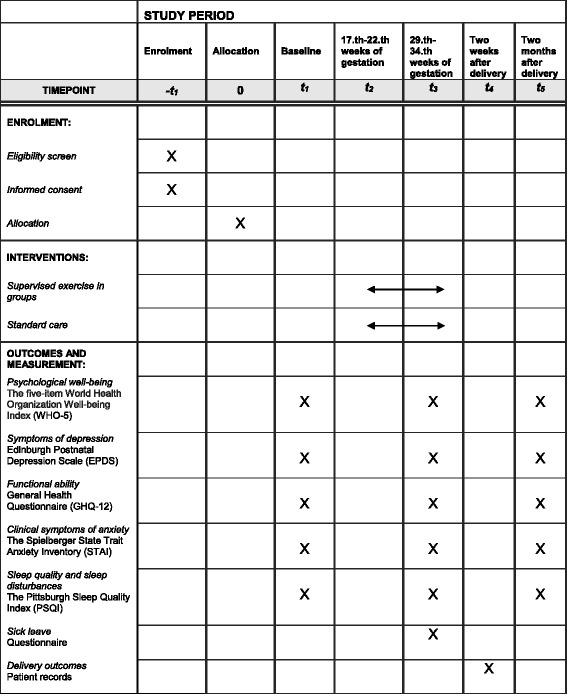
Consolidated Standards of Reporting Trials (CONSORT) showing time points of enrollment, intervention, and outcome measures

Data will be obtained at baseline and four times during the study period: at 17–22 weeks of gestation (T1), at 29–34 weeks of gestation (T2), 2 weeks after delivery (T3), and 2 months after delivery (T4). The SPIRIT figure (*see* Fig. 2) provides an overview of the outcome measures used in the trial and their time points. The participants will fill out the first electronic questionnaire at the face-to-face meeting where randomization takes place, and they will receive a link to the two following questionnaires (T2, T4) by email. Prior to the data collection, an email is sent to the participants reminding them of the upcoming data collection.

#### Primary outcome

Psychological well-being, the primary outcome, will be measured by the WHO-5 score. The WHO-5 is a short generic rating scale measuring general subjective current (the last 2 weeks) psychological well-being [27]. The questionnaire has five items and covers positive mood (feeling in good spirits), vitality (being active and waking up feeling refreshed and well-rested), and being interested in things [33]. It contains five positively phrased items; each item is scored on a Likert scale (0 = none of the time and 5 = all of the time). The raw score ranges from 0 (absence of well-being) to 25 (maximal well-being) and is multiplied by a factor of 4 to give a scale ranging from 0 to 100, where a score of 0 is the lowest possible well-being and a score of 100 is the highest possible well-being [27]. WHO-5 has high clinimetric validity, and it can be used as an outcome measure in clinical trials in addition to a clinical screening tool for depression [27].

#### Secondary outcomes

Five secondary outcomes will be measured:

1. *Symptoms of depression*, defined as a cutoff score ≥13 and a cutoff score ≥10 measured by the Edinburgh Postnatal Depression Scale (EPDS). EPDS is a self-reported questionnaire measuring depression and consisting of 10 items scored on a 4-point Likert scale (0–3). The scale addresses the intensity of common depressive symptoms within the previous 7 days, where 0 indicates absence of depressed mood and 3 indicates the worst mood. The lowest score is 0, and the highest score is 30. According to some literature, a cutoff score ≥13 indicates an increased likelihood of clinical depression [34, 35], whereas other studies suggest a cutoff score ≥10 to minimize the risk of failed detection of cases [35]. To meet the findings in previous studies, we will use two cutoff scores, ≥13 and ≥10, in our statistical analysis. The EPDS has been validated for the detection of antenatal and postpartum depression [34–36] and has previously been translated into Danish and then back-translated to check that the questions elicited the intended information [37].
2. *Functional ability*, measured by the 12-item General Health Questionnaire (GHQ-12). The GHQ-12 is used to measure psychological well-being in adults over the previous 2 weeks [38]. The GHQ-12 screens for symptoms common to any nonpsychotic disorders and is focused on two areas: the inability to carry out normal functions and the appearance of new and distressing psychological phenomena [38]. The GHQ-12 contains 12 items, and the total score of the GHQ-12 varies from 0 to 36 using a 4-point Likert scale (0–3) [39].
3. *Clinical symptoms of anxiety*, measured by the State-Trait Anxiety Inventory (STAI). The STAI has previously been used for pregnant women [40]. It consists of 40 items, but only the state scale of the inventory will be used in this study, because the state scale measures symptoms of anxiety as anxiety is defined according to DSM-5 [41]. The 20 items concerning state anxiety are scored on a 4-point Likert scale and indicate how well the participant is feeling today (1 = not at all, 2 = somewhat, 3 = moderately so, and 4 = very much so). The total score for state anxiety ranges from 20 to 80. This inventory has been reported to have a high degree of internal consistency and validity [42]. We have chosen to cite the original version of the STAI because this is recommended by leading Danish experts in the field.
4. *Sleep quality and sleep disturbances*, measured by the Pittsburgh Sleep Quality Index (PSQI). The PSQI is a subjective scale measuring sleep quality and sleep disturbances over a 1-month period [43]. The scale addresses quantitative aspects of sleep, such as sleep latency and sleep duration, as well as more subjective aspects, such as restfulness of sleep [43]. Overall sleep quality is measured by means of 19 questions, and the total score is based on 7 “component” scores, each of which has a range of 0–3 points. A score of 0 indicates no difficulty, whereas a score of 3 indicates severe difficulty. The sum of the component scores results in a “global” score ranging from 0 to 21 points, where 21 indicates severe sleep problems. The PSQI has been found to be useful in pregnancy research, and good internal consistency and construct validity have been reported [44].
5. *Pregnancy and delivery outcomes*, comprising sick leave (measured in days), hospitalization (measured in number of hospital admissions and days), labor onset (spontaneous or induced), use of epidural anesthesia during delivery (yes or no), duration of labor (measured in hours), mode of delivery (percentage of participants with spontaneous delivery, vacuum extraction, or cesarean section), birthweight (measured in kilograms), and birth length (measured in centimeters). This information will be obtained from patient records.

### Data analysis

The primary data analysis will be performed on the basis of the intention-to-treat principle. We will compare baseline data for the two groups with Student’s *t* test, the chi-square test, or nonparametric tests. It is anticipated that a large proportion of values for the outcome variable will be missing, owing to dropout and nonresponse to the questionnaire. These data will be assumed missing at random, and therefore observed patient characteristics will be used to impute missing data by means of multiple imputation. Because it is expected that there will be a dropout rate of 13% (20 women) in each group and that some women will participate in the group training only a few times, in addition to the intention-to-treat analyses, we will perform a per-protocol analysis of the women who perform ≥75% of all training sessions.

### Pilot study

Before initiating a full-scale trial, a pilot study with 20 participants was conducted from April to June 2016. This was done to examine the recruitment procedure, dropout rates, and acceptability of and compliance with the exercise program and the questionnaires [45]. The experiences of the participants and key health care professionals involved in the project were registered and evaluated. The proportion of pregnant women eligible for participation in the study and who wished to participate were as expected, and we therefore expect to be able to recruit the needed 300 participants according to the study power calculation. Compliance among the nine pregnant women in the intervention group participating in the pilot study was acceptable. The pilot study led to minor changes. Hand grip strength measurement (by North Coast Hydraulic Hand Dynamometer) was included at baseline to provide an objective measure of total muscle strength [26]. The STAI was added to the questionnaires because a large proportion of the participants had anxiety prior to pregnancy; use of a specific measure of anxiety is therefore relevant. Furthermore, the PSQI was added to the questionnaires because participants reported that their quality of sleep had improved during the intervention period. Finally, at the request of the participants, the exercise intervention was adjusted so that it is possible to choose between treadmills and cross-trainers in addition to exercise bikes during the endurance training part of the intervention, instead of exercise bikes being the only option.

## Discussion

Antenatal depression and anxiety are associated with adverse complications such as preterm birth, which is a leading cause of infant morbidity [6], and result in emotional suffering for many pregnant women. The antenatal period theoretically offers an opportunity to implement preventive and effective interventions for pregnant women with mental illness and thereby reduce adverse outcomes and promote health for women and children. The EWE Study is therefore a highly relevant study and is, to our knowledge, the largest RCT designed to evaluate the effectiveness of supervised exercise in groups for improving psychological well-being among pregnant women at risk of depression. In addition to the large sample size, postnatal follow-up data will be provided, intention-to-treat analysis will be carried out, and the statistician performing the statistical analyses will be blinded to the allocation to the intervention or control group. Furthermore, baseline data will be collected to allow a comparison of the two groups, attendance at exercise classes will be documented, and data on safety and adverse events in the intervention group will be reported. These elements have been pointed out by authors of a meta-analysis as missing in previous RCTs on the topic [20]. The strongest risk factor for depression and anxiety during pregnancy or after delivery is a history of depression or anxiety prior to pregnancy [16]. It is therefore relevant to look at both diagnoses to improve psychological well-being among pregnant women at risk of depression. One study showed that depressive disorders and anxiety can be accompanied by a tendency for passivity and withdrawal, which may affect compliance [46]. However, a Danish study showed that the pregnant population attending Rigshospitalet, Copenhagen University Hospital, has a high proportion of well-educated women [47], who are associated with a high engagement in exercise [47]; therefore, we expect that compliance is in line with our expectations regarding this study population. This was also supported by the experiences in the pilot study. If proven effective, the intervention will offer a way for pregnant women at risk of depression or anxiety to actively do something to increase their physical and psychological well-being. The intervention is expected to be acceptable, feasible, cost-effective, and applicable, as well as being complementary to the existing antenatal care provided for this group of women.

To conclude, we have described a protocol for an RCT aimed at evaluating whether supervised exercise in groups for pregnant women with a current or previous history of depression and/or anxiety improves psychological well-being during pregnancy. The results will provide evidence useful for the organization of care for pregnant women and their families.

### Trial status

Participants are currently being recruited into the EWE Study. The recruitment period is estimated to run from July 2016 to December 2018, with follow-up until March 2019.

## Additional file

Additional file 1: SPIRIT checklist. (DOC 123 kb)

## Abbreviations

CONSORT: Consolidated Standards of Reporting Trials
DSM-5: *Diagnostic and Statistical Manual of Mental Disorders, Fifth Edition*
EPDS: Edinburgh Postnatal Depression Scale
EWE: Effect of Group Exercise on Mental Wellbeing Among Pregnant Women at Risk of Perinatal Depression trial
GHQ-12: General Health Questionnaire
PSQI: Pittsburgh Sleep Quality Index
RCT: Randomized controlled trial
SPIRIT: Standard Protocol Items: Recommendations for Interventional Trials
STAI: State-Trait Anxiety Inventory
WHO: World Health Organization
WHO-5: WHO (Five) Well-being Index
